# Evaluation of a novel dipotassium phosphate bolus for treatment of metabolic disorders in dairy cattle

**DOI:** 10.3389/fvets.2023.1274183

**Published:** 2023-12-08

**Authors:** Walter Verhoef, Sjoert Zuidhof, Joseph A. Ross, Kendall Beaugrand, Merle Olson

**Affiliations:** ^1^Alberta Veterinary Laboratories Ltd., Calgary, AB, Canada; ^2^Sjoert Zuidhof Consulting, Okotoks, AB, Canada; ^3^Chinook Contract Research Inc., Airdrie, AB, Canada; ^4^Alberta Veterinary Laboratories Ltd., Calgary, AB, Canada

**Keywords:** dipotassium phosphate, K Phos-Boost, downer cows, hypophosphatemia, hypokalemia, ketosis, periparturient

## Abstract

A dipotassium phosphate bolus (K Phos-Boost) has been developed to treat both hypophosphatemia and hypokalemia, as the clinical signs of both conditions are similar and occur in the early post-partum period. The objective of this research was to evaluate the efficacy and application of the bolus for prevention and treatment of metabolic diseases that are common in dairy production systems. *Study 1 (Pharmacokinetic study)*: Healthy post-partum cows were either untreated or received two K Phos-Boost boluses at times 0, 24, and 48 h. Blood was taken at *t* = 0, 2-, 4-, 6-, 8-, 10-, 24-, and 52-h post-treatment for analysis of total serum minerals. There was an increase in serum phosphorous to normal levels within 2 h of treatment with the bolus, but control cows remained hypophosphatemic. Serum potassium was significantly elevated 2 h after bolus administration relative to control, while calcium, magnesium, sodium, and chloride levels were not affected by the K Phos-Boost bolus. *Study 2 (Downer Cow Treatment)*: K Phos-Boost boluses were provided to cows that were unresponsive to intravenous calcium therapy and had been unable to stand for over 24 h (“downer cows”). Most cows (16 of 19) treated with two boluses were standing without assistance between 1 and 24 h after treatment and the serum phosphorous was increased to normal levels in five of five tested animals. *Study 3 (Ketosis Treatment)*: cows with clinical ketosis were provided with propylene glycol and K Phos-Boost boluses (*n* = 29) or only propylene glycol (*n* = 23). Cows treated with the K Phos-Boost bolus showed a more rapid recovery by increased milk production (3.9 kg/day) and rumination rate (97 min/day). *Study 4 (Health Promotion)*: cows in herds with >40% post-partum hypophosphatemia received K Phos-Boost boluses (*n* = 130) or no treatment (*n* = 146) following calving. There was a trend for treated 2^nd^-lactation animals to have higher milk production after 30 DIM (49.1 vs. 46.2 kg/day; *P* = 0.09). There were no significant differences between control and bolus treated animals in the incidence of subclinical ketosis, post-calving total health events, or culling rates. The K Phos-Boost bolus is a novel product and has the potential to treat and prevent several important metabolic diseases in dairy cattle. The studies described in this paper are early investigations and further research should be conducted to demonstrate the applications of a dipotassium phosphate bolus in dairy cows.

## 1 Introduction

It is recognized that inadequate blood phosphorus and potassium is associated with downer cows as well as reduced feed intake, reduced rumen and intestinal motility, and an increased susceptibility to other metabolic and infectious diseases (1). Early treatment of such metabolic disorders is beneficial for both positive clinical outcomes and economic returns (2, 3). The incidence of periparturient hypophosphatemia in dairy cattle has been reported to be 50% on the day of parturition and 10%−15% during the first 2 weeks post-partum (4). It has been shown that periparturient supplementation of phosphate in the ration does not prevent post-parturient hypophosphatemia (5), so phosphate deficiencies must be addressed at the time of calving. Subnormal serum or plasma phosphorous levels have been associated with downer cow syndrome and alert downer cows following treatment for hypocalcaemia (milk fever). Hypophosphatemia is also associated with hemoglobinuria, incoordination, reduced milk production, reduced feed intake, and increased risk of post-partum disease (mastitis, ketosis, abomasum displacement, and abomasal volvulus) (1, 4, 5). Intravenous administration of phosphate salts has been shown to provide only transient correction of hypophosphatemia (2–4 h) and is therefore an unsuitable treatment for hypophosphatemia, which can persist for several days during the post-partum period (4). Oral phosphate drenches with phosphate salts, sodium phosphate (NaH_2_PO_4_) or disodium phosphate (Na_2_HPO_4_) equivalent to 60 g of phosphorus have been used to correct hypophosphatemia for at least 24 h (6–8). Longer term correction for cows may require daily drenching with a gastric tube, as a diet adjustment for the entire group to which the cow belongs is not practical. Repeated drenching is stressful to the cow and is labor intensive. A novel bolus containing phosphate salts may be an appropriate alternative.

Cows receive a potassium-rich diet and readily excrete excessive potassium through the kidney (9). The normal physiological blood plasma potassium levels of a cow ranges between 3.9 and 5.8 mmol/L, however the ratio of the intracellular to the extra cellular potassium contents are more relevant (9–12). A sudden decrease in food intake can result in hypokalemia, as the kidneys may not be able to respond to the decrease in dietary potassium (10–12). Hypokalemia is also associated with retained placenta, clinical mastitis and abomasal displacement (10–12). The clinical signs of hypokalemia are muscle weakness and recumbency (1, 10–12). Hypokalemia has been treated with oral drenching of potassium chloride (KCl) since intravenous therapy carries a risk of cardiac arrest (11). The optimum amount to be delivered per day has been reported to be 60–250 g/100 kg body weight, although most practitioners recommend 250 g per cow per day for 3–5 days (11).

There has been limited research into prevention and treatment of post-parturient hypophosphatemia and hypokalemia. One of the reasons is that a readily available and easy to deliver therapeutic product has not been available. A dipotassium phosphate (K_2_PO_4_) bolus (“K Phos-Boost”) has been developed by Solvet Animal Health (Calgary, Alberta, Canada) to treat both hypophosphatemia and hypokalemia, as the clinical signs of both conditions are similar and occur in the early post-partum period. Each K Phos-Boost Bolus (Solvet Animal Health, Calgary, Alberta, Canada) weighs 230 g and consists of dipotassium phosphate (K_2_HPO_4_), providing 100 g of phosphate and 83 g of potassium per bolus. Each bolus is individually vacuum sealed, and the bolus retains its shape under storage temperatures below 40°C (unpublished results). The bolus completely dissolves in the rumen within 30 min (based on unpublished *in vivo* studies using fistulated cattle). The recommended published dose for phosphorous and potassium deficient dairy cows is 198 g of phosphate and 131 g of potassium per day for 1–5 days (4, 11). When two dipotassium phosphate boluses are provided, cows receive 200 g of phosphate and 166 g of potassium per day. This closely matches the published recommended daily dosages. The suggested dose is therefore two boluses, once per day, for up to 3 days.

The rationale for providing a bolus that supplements both potassium and phosphorous is that, although hypophosphatemia is easy to identify in post-partum dairy cattle using blood levels, hypokalemia is not (5). A serum potassium concentration of <2.5 mEq/L indicates severe hypokalemia which occurs after intracellular potassium is depleted. Early lactation cows are reported to be in a negative potassium balance as they excrete a significant amount of potassium in the milk (13). If the cow is not deficient in intracellular potassium, it is able to readily eliminate the excess potassium in the urine (9, 12).

This paper provides early studies investigating the biochemical, pharmacological, and clinical effects of a dipotassium phosphate (K Phos-Boost) bolus in post-partum dairy cows where hypophosphatemia and hypokalemia are most common.

## 2 Materials and methods

### 2.1 Pharmacokinetics in post-partum dairy cows

A 600-cow Holstein dairy herd in the province of Alberta, Canada was selected for the pharmacokinetic study where cows were milked in a rotary milking system three times daily. The post-partum ration consisted of 7.85 kg of grass silage, 8 kg of corn silage, 3 kg of dry hay, and 4 kg of a supplement (calcium 0.56% D.M., phosphorus 0.31% D.M., magnesium 0.50% D.M., and potassium 1.41% D.M.). Only clinically healthy cows in their second and third lactations were enrolled in this study and were allocated using a randomization chart to one of two treatment groups: A = Two K Phos-Boost boluses at times 0, 24, and 48 h (i.e., treated group, *n* = 4); B = Untreated (i.e., control group, *n* = 5). Blood was taken from the coccygeal vein pre-treatment at time 0 (time of first treatment) and at 2, 4, 6, 8, 10, 24 (time of second treatment), 30, 48 (time of third treatment), and 52 h. A *post-hoc* power calculation at the 2-h time point indicated that, at α = 0.05, power = 92.9% and 99.3% for P and K, respectively. The first (*t* = 0) bolus administration was within 12 h of calving. The blood was immediately centrifuged on the farm (3,000 rpm, 10 min) and the serum transferred into micro-centrifuge tubes for storage at −18°C and transport to the laboratory, where total serum phosphorous, potassium, calcium, magnesium, sodium, and chloride were analyzed on an Element DC5x Veterinary Chemistry Analyzer (HESKA, Barrie, Ontario, Canada).

### 2.2 Treatment of hypophosphatemic downer cows

The hypothesis was that cows that have been unable to stand for over 24 h, after going down with hypophosphatemia, or hypophosphatemia and hypocalcemia, will respond to treatment by increasing serum phosphorous and intracellular potassium. Veterinary practitioners in the provinces of Ontario and Quebec were provided K Phos-Boost boluses and enrolled cases of post-partum cows that met the criteria of a downer for over 24 h that were refractory to intravenous calcium treatments and in sternal recumbency. Seventeen cows that were downers for ~24 h and two cows that were down for ~48 h were enrolled into the study. Blood was collected from the coccygeal vein and serum calcium and phosphorous was measured before treatment with the K Phos-Boost Bolus. Cows received two K Phos-Boost boluses daily until they were standing (maximum 3 days of treatment). Hypocalcemic cows also received intravenous calcium borogluconate. Serum calcium and phosphorous were measured ~24 h following K Phos-Boost Bolus treatment in only 5 of the 19 cases. Response to treatment was considered a recovery when the cow stood and did not revert to the downer state. There were no untreated controls in this study as it was considered unethical not to treat downer cows. It is well-known that post-partum cows that have been recumbent for over 24 h have very poor outcomes (14).

### 2.3 Treatment of clinical ketosis

The study hypothesis was that phosphorous and potassium supplementation using dipotassium phosphate bolus will stimulate appetite and improve recovery from the ketotic state in dairy cows. The study was conducted with cows from nine commercial dairy facilities in Quebec, Canada. Cows that exhibited decreased milk production, reduced feed intake, and depression were subjected to a blood ketone test (Precision Xtra™ meter using the Precision Xtra™ Blood Ketone test strips, Abbott Laboratories, Abbott Park, IL). Cows with a ketone level ≥1.4 mM BHB (beta hydroxybutyrate) were enrolled in the study and randomly allocated to one of two treatment groups: A = two dipotassium phosphate boluses immediately following the diagnosis of ketosis and for two more consecutive days (treatment group; *n* = 29); *B* = no bolus (control group; *n* = 23). All cows received the treatment for clinical ketosis according to the site protocol which included intravenous dextrose and oral propylene glycol. Daily rumination and milk production data were collected from 2 days prior to the ketosis diagnosis through to 5 days post-diagnosis. Ketone levels were checked again at Day 5.

### 2.4 Treatment of hypophosphatemia within 12 h after calving

The hypothesis of the study was that post-partum phosphorous supplementation in herds with hypophosphatemia will stimulate appetite and recovery from calving in the transition period. This will increase milk production and decrease post-partum health events. As hypocalcemia is common in post-partum cows, supplementing cows with calcium after calving is often a routine practice. A total of 276 Holstein dairy cows, in their second lactation and greater, from four dairy herds were enrolled. Enrolled herds were located in the provinces of Alberta (two herds), Ontario (one herd), and Québec (one herd). In these herds, 50% of cows were below the 1.3 mmol/L study selection threshold, with phosphorous levels varying from 0.66 to 2.16 mmol/L. The Phosphorous was analyzed on an Element DC5x Veterinary Chemistry Analyzer (HESKA, Barrie, Ontario, Canada). All cows that calved in both the treated and control groups received 2 calcium boluses (Cal-Boost, Solvet, Calgary, AB) within 12 h of calving. Even numbered cows received an additional 2 boluses of K Phos-Boost (Treatment) while odd numbered cows did not receive the K Phos-Boost boluses (Control). All cows were tested for subclinical ketosis between 7 and 14 days in milk using blood taken from the coccygeal vein for evaluation of ketone levels (Freestyle Precision Xtra ketone strip, Abbott Labs). Animals with a blood ketone result of 1.2 mmol/L or greater were considered to have subclinical ketosis. Health events (milk fever, retained placenta, clinical ketosis, displaced abomasum, mastitis, respiratory disease) were recorded for the first 60 days in milk. Milk production on days 30 and 60 as well as peak milk was obtained from the dairy herd management software.

### 2.5 Statistical analyses

Statistical analyses were performed essentially as described elsewhere (15, 16). The experimental unit was defined as each individual animal. Health events for mildly hypophosphatemic cows were analyzed by Fisher's Exact Test. BHB data for ketotic cows was analyzed using a two-tailed, unpaired *T*-test; normality was confirmed with a Shapiro-Wilk test. All other data were analyzed using a mixed-effects model, wherein time, treatment, and the time-by-treatment interaction effects were considered fixed, while animal and residual effects were considered random. In all cases, the significance level was *P* < 0.05, while a trend was defined as a *P*-value between 0.05 and 1.0. Statistical analyses were conducted in Prism v 9.5.1 (GraphPad Software, San Diego, CA, USA).

### 2.6 Ethics approval and consent

The present field-based study was conducted in compliance with the best practice of veterinary care in accordance with the research guidelines set forth by the Canadian Council on Animal Care and each study was reviewed by an animal ethics committee. The owners of the cattle provided informed consent for their animals to be used in the present study.

## 3 Results

### 3.1 Study 1: pharmacokinetics in post-partum dairy cows

**Figure 1:**
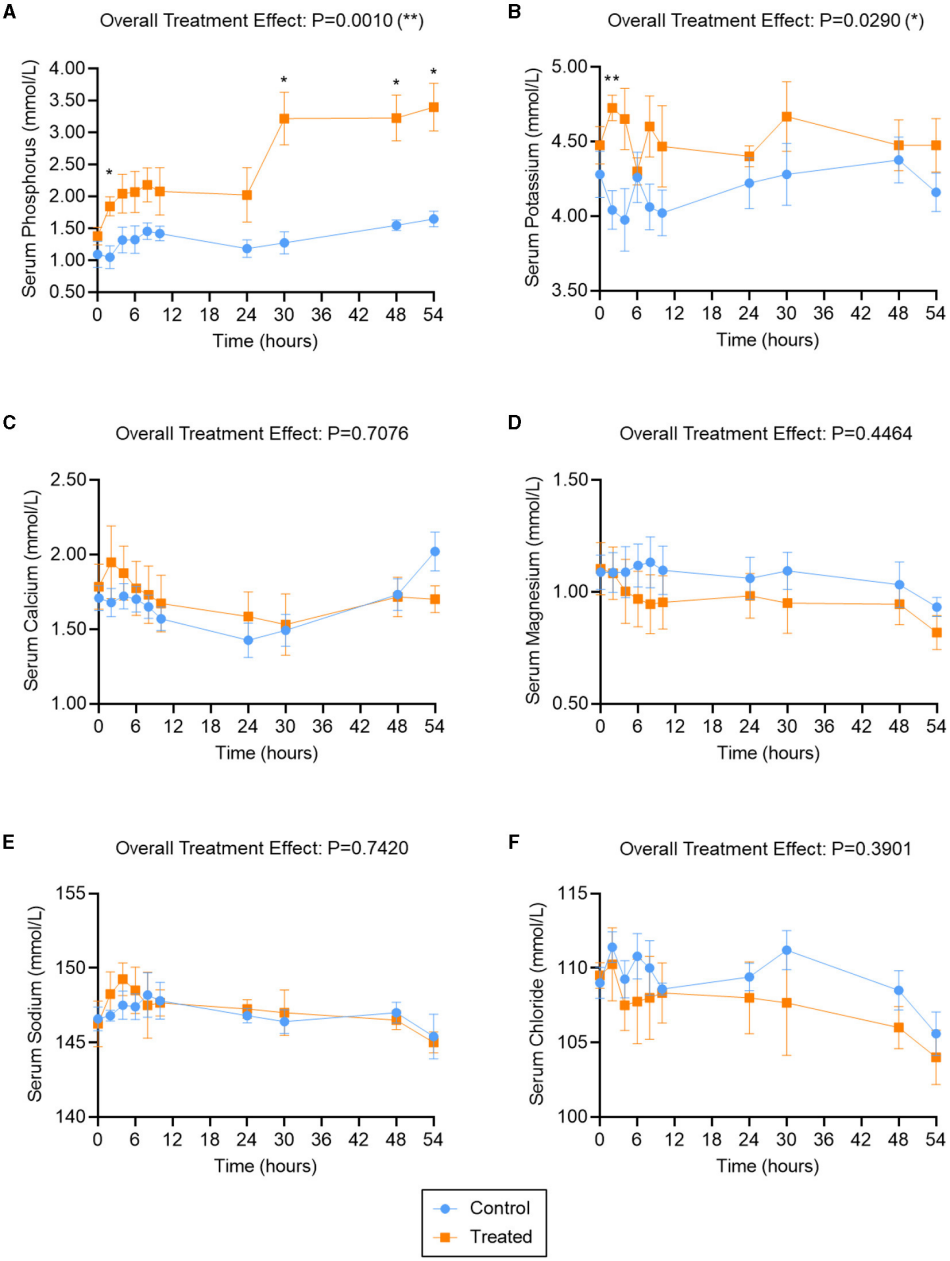
Serum ion levels in second- and third-lactation cows treated with K Phos-Boost boluses (Treated) vs. untreated (Control). Serum Phosphorous **(A)**, Potassium **(B)**, Calcium **(C)**, Magnesium **(D)**, Sodium **(E)**, or Chloride **(F)** were measured at the indicated time points; two boluses were administered to the Treated group at times 0, 24, and 48 h. Bars represent the mean ± SEM (Standard Error of the Mean) for 5 (Control) or 4 (Treated) animals. Data were analyzed for statistical significance using a mixed model with repeated measures. *P < 0.05; **P < 0.01.

The serum phosphorus, potassium, calcium, magnesium, sodium, and chloride levels in treated cows (two K Phos-Boost boluses at times 0, 24, and 48 h) and control (untreated control cows) are provided in Figure 1. Overall, a significant (*P* = 0.001) increase in serum phosphorous was observed in bolus-treated cows, relative to untreated cows, over the 54-h time course (Figure 1A). The increase in serum phosphorous was significant as early as 2 h after administration of the first boluses and increased sharply after administration of the second set of boluses (at *t* = 24 h), and was significantly elevated, relative to untreated animals, at *t* = 30, 48, and 54 h (Figure 1A). Serum potassium levels were also significantly elevated overall (*P* = 0.029) in bolus-treated vs. untreated cows, with a sharp and significant increase at *t* = 2 h after administration of the first boluses (Figure 1B). Potassium levels did not remain elevated (compare Figures 1A, B) and returned to normal levels within 12 h. Subsequent treatments with the K Phos-Boost bolus did not yield a significant increase in potassium levels 6 h after receiving the bolus. None of the other ions tested (calcium, magnesium, sodium, or chloride) were significantly different in bolus-treated vs. untreated cows over the 54-h time course (Figures 1C–F).

### 3.2 Study 2: treatment of hypophosphatemic downer cows

The study yielded data for 19 Holstein cows from 17 different herds (Table 1). Blood samples collected immediately before K Phos-Boost bolus treatment indicated that 7 of the 19 cows were hypophosphatemic (serum phosphorus <1.30 mmol/L) with normal serum calcium (calcium >2.12 mmol/L), and 12 of the cows were both hypophosphatemic (phosphorus <1.30 mmol/L) and hypocalcemic (calcium <2.12 mmol/L). Sixteen cows fully recovered and were standing between 1 and 24 h following treatment with two K Phos-Boost boluses. The serum phosphorous and calcium were collected 24 h after treatment with K Phos-Boost boluses in five cows and demonstrated that serum phosphorus was normal (4 out of 5 cows) or returning to normal (one out of five cows). Three cows (13, 14, and 15) remained as downer cows and were euthanized. Two of the cows that died (13 and 14) had been downers for over 2 days despite having normal serum calcium levels but were hypophosphatemic. Cow 15 was both hypophosphatemic and hypocalcemic but did not respond to calcium and phosphorous supplementation.

**Table 1:** Effects of K Phos-Boost bolus treatment on downer cows.

**Case**	**P (mmol/L) (*T* = 0 h)**	**P (mmol/L) (*T* = 24 h)**	**Ca (mmol/L) (*T* = 0 h)**	**Ca (mmol/L) (*T* = 24 h)**	**Outcome**	**P and Ca status**
1	1.25	N/A	2.40	N/A	Recovered	HypoP
2	1.00	N/A	1.26	N/A	Recovered	HypoP + HyoCa
3	0.68	N/A	2.61	N/A	Recovered	HypoP
4	1.14	N/A	1.53	N/A	Recovered	HypoP + HyoCa
5	0.85	N/A	0.78	N/A	Recovered	HypoP + HyoCa
6	0.95	N/A	2.47	N/A	Recovered	HypoP
7	0.72	3.29	1.00	1.53	Recovered	HypoP + HyoCa
8	1.06	N/A	1.29	N/A	Recovered	HypoP + HyoCa
9	0.88	N/A	1.86	N/A	Recovered	HypoP + HyoCa
10	0.61	N/A	0.77	N/A	Recovered	HypoP + HyoCa
11	0.43	N/A	1.10	N/A	Recovered	HypoP + HyoCa
12	0.70	N/A	2.95	N/A	Recovered	HypoP
13	1.20	N/A	2.30	N/A	Deceased	HypoP
14	0.79	1.09	2.81	2.18	Deceased	HypoP
15	0.63	N/A	2.11	N/A	Deceased	HypoP + HyoCa
16	0.54	3.02	1.22	2.58	Recovered	HypoP + HyoCa
17	1.23	2.02	2.17	2.30	Recovered	HypoP
18	0.20	2.61	0.83	2.51	Recovered	HypoP + HyoCa
19	0.79	N/A	1.32	N/A	Recovered	HypoP + HyoCa

### 3.3 Study 3: effects of the K Phos-Boost bolus on rumination, milk production, and ketosis in cows with clinical ketosis

**Figure 2:**
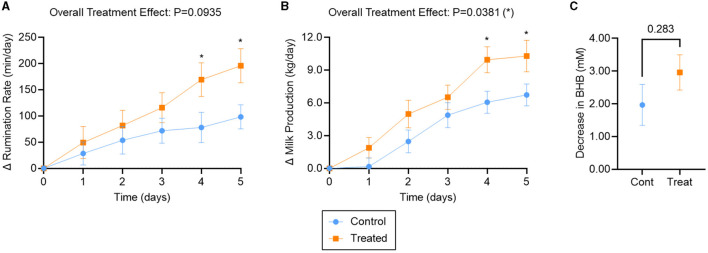
Outcomes for ketotic cows treated with K Phos-Boost boluses (Treated) vs. untreated (Control). **(A)** Increase in Rumination Rate (min/day) relative to Day 0, **(B)** increase in Milk Production (kg/day) relative to Day 0, **(C)** decrease (by Day 5) in serum Beta-Hydroxybutyrate (mM) relative to Day 0. Two boluses were administered to the Treated group at times 0, 24, and 48 h. Bars represent the mean ± SEM (Standard Error of the Mean). For **(A)**, *n* = 22 (Control) or 27 (Treated); for **(B)**, *n* = 23 (Control) or 29 (Treated) animals. A subset of those animals was examined for BHB **(C)**, such that *n* = 9 (Control) or 20 (Treated). Data in **(A)** and **(B)** were analyzed for statistical significance using a mixed model with repeated measures; data in **(C)** were analyzed with an unpaired, two-tailed *T*-test. *P < 0.05.

No statistically significant differences in rumination rate, milk production, and BHB were observed between treatment and control groups at day 0 (Supplemental Figure S1). The change in rumination rate, milk production, and BHB, relative to their respective day 0 levels, are reported for each treatment group in Figure 2.

The rumination rate increased for both treatment groups over the 5-day observation period (Figure 2A). Overall, mixed effects analysis indicated that rumination tended to be improved in dipotassium phosphate bolus-treated vs. untreated animals over the observation period (treatment effect: *P* = 0.09). Rumination rate was significantly different between treatments and controls on day 4 (91 min/day; *P* = 0.04) and day 5 (97 min/day; *P* = 0.02; Figure 2A).

Milk production also increased for both treatment groups over the 5-day observation period (Figure 2B). Overall, milk production was significantly improved in bolus-treated vs. untreated animals (treatment effect: *P* = 0.04), with significantly higher milk production by days 4 (3.9 kg/day; *P* = 0.02) and 5 (3.6 kg/day; *P* = 0.04; Figure 2B).

BHB levels decreased back to normal levels by Day 5 for both treatment groups (Supplemental Figure S1C). No statistically significant difference in BHB was observed between bolus-treated and untreated animals over the 5-day observation period (*P* = 0.28; Figure 2C).

### 3.4 Study 4: effects of the bolus on subclinical ketosis, milk production, and post-parturient health events when administered to hypophosphatemic herds within 12 h after calving

Relative to the control group, dipotassium phosphate supplemented cows had a lower incidence of subclinical ketosis (9.5% vs. 14.4%) and post-calving total health events (18.5% vs. 23.3%), although these did not reach statistical significance (*P* = 0.27 and 0.38, respectively). Control and treated animals also had a statistically similar culling rate (4.6% vs. 2.1%, *P* = 0.31; Table 2).

**Table 2:** Health events for mildly hypophosphatemic cows treated with K Phos-Boost at calving vs. untreated controls.

**Event**	**Control**	**K Phos-Boost**	***P*-value^a^**
SCK	21/146 (14.4%)	12/126 (9.5%)	0.27
Health Event	34/146 (23.3%)	24/130 (18.5%)	0.38
Culled/Died	3/146 (2.1%)	6/130 (4.6%)	0.31

^a^*P*-values were determined using Fisher's Exact Test.

**Figure 3:**
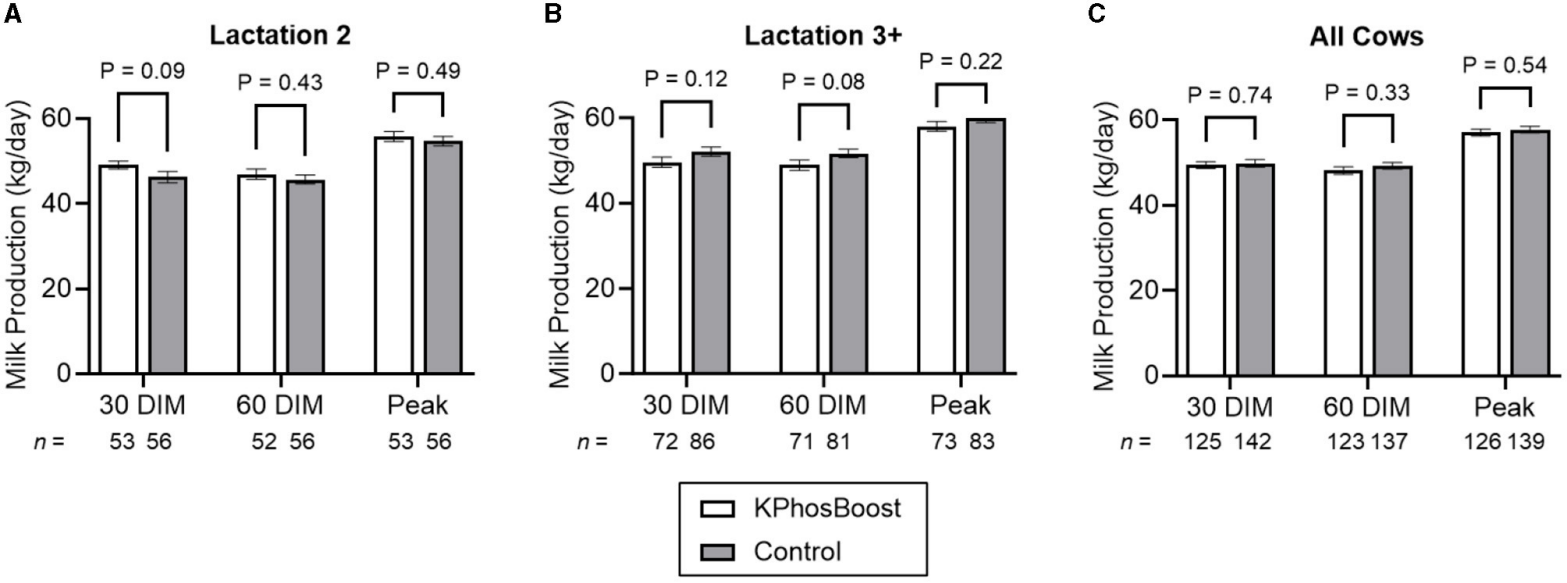
Milk production in hypophosphatemic cows treated with K Phos-Boost, vs. untreated Controls, within 12 h of calving. **(A)** Second lactation animals only. **(B)** Animals in their third or higher lactation. **(C)** All animals. Bars represent the mean ± SEM (Standard Error of the Mean) for the indicated number of animals. Data were analyzed for statistical significance using an unpaired, two-tailed *T*-test.

Overall, there was no statistically significant difference in milk production between the two groups (Figure 3), although 2nd-lactation animals tended to have higher production after 30 days in milk (DIM) for the treated vs. control group (mean: 49.1 vs. 46.2 kg/day; SEM: 0.9 vs. 1.4 kg/day; *P* = 0.09; Figure 3A).

## 4 Discussion

Metabolic disorders are associated with disturbances of one or more blood metabolites of cattle which result in a group of diseases that most commonly affect dairy cows during the first month after parturition (2, 3, 17). These diseases include milk fever, downer cows, retained placenta, ketosis, left displaced abomasum, rumen acidosis, laminitis, liver abscesses, and bloat. There is a strong association of these diseases with each other, although the mechanisms are not well understood. Approximately 75% of the metabolic diseases occur within the periparturient period (3 weeks before calving and 3 weeks after calving). This period is associated with decrease serum concentrations of macrominerals and glucose (1, 18).

It has been suggested that approaches to metabolic diseases need to be updated because of new findings about these disorders (2). The studies presented in this paper demonstrate the need for further research in post-parturient phosphorus and potassium supplementation to address metabolic diseases in dairy cattle. The dipotassium phosphate bolus provides a convenient tool to further investigate the benefits of oral supplementation of potassium and phosphorus.

### 4.1 Pharmacokinetics

Within 2 h of treatment with the potassium phosphate bolus (62 g of phosphorus), serum phosphorus was significantly increased compared to controls (Figure 1A) and within the normal range (>1.3 mmol/L). This rapid response is similar to that observed when cattle were drenched with 60 g of phosphorous as sodium phosphate (NaH_2_PO_4_), disodium phosphate (Na_2_HPO_4_), or mono potassium phosphate (KH_2_PO_4_) (6– 8). Previous studies have shown that calcium phosphate (CaHPO_4_) or magnesium phosphate (MgHPO_4_) had a delayed and less pronounced change in serum phosphorus compared to corresponding sodium or potassium salts (6–8). The dipotassium phosphate boluses maintained an elevated serum phosphorous for over 24 h and a second treatment with 2 boluses increased these levels from 2.0 mmol/L to 3.0 mmol/L. The third treatment at 48 h did not elicit a significant increase in serum phosphorous. This study provides evidence that the dipotassium phosphate bolus provides the necessary phosphorous supplementation to address the hypophosphatemic state in dairy cows.

Treatment with two dipotassium phosphate boluses (166 g of potassium) rapidly increased serum potassium within 2 h of administration. This increase was transient, and the serum potassium was at pre-treatment levels within 6 h of administration. A similar response was elicited following the 24- and 48-h treatments. The reduction in serum potassium is most likely associated with either movement of potassium into the intracellular compartment, or it being eliminated in the urine or in the milk. Oral administration of potassium chloride or potassium propionate has shown a similar rapid increase in serum potassium with peak levels at ~6 h (10, 12). It was shown that there was no difference in serum response when administration of potassium chloride at 0.5 g/kg BW was delivered as multiple small doses or two individual doses (10). This suggests that a single daily treatment with the dipotassium phosphate bolus meets the daily potassium requirements.

There is a concern that oral phosphate supplementation may cause transient hypocalcemia and hypomagnesemia due to interference of rumen absorption of minerals (2, 5, 10, 19, 20). In this study, there was no significant change in serum calcium or magnesium up to 5 days following treatment with dipotassium phosphate bolus. There was probably insufficient phosphate in the bolus or the duration of treatment was too short to cause hypocalcaemia or hypomagnesemia.

### 4.2 Treatment of downer cows

Both veterinarians and producers are frustrated that, despite treatment, downer cows are often still unable to get up. Parturient paresis is common in high-producing dairy cows. Affected cows are frequently hypocalcemic and hypophosphatemic. Most cattle respond clinically by standing following intravenous and/or subcutaneously administered calcium borogluconate. However, some cows do not respond and are defined as downer cows. Cows that are unable to stand with normal serum calcium (>2.12 mmol/L) but low serum phosphorus (<1.3 mmol/L) are referred to as alert downer cows. In this study, we defined downer cow syndrome as “a cow that is in sternal recumbency for more than 24 h that is bright and alert.” Downer cow syndrome has been poorly understood and may be associated with hypophosphatemia and hypokalemia that results in muscle weakness, muscle necrosis and myoglobinuria (2, 14, 21). Phosphorous is necessary for the synthesis of adenosine triphosphate for membrane integrity and a source of energy (1, 2, 4). Potassium is a principal ion involved in maintenance of muscle function and nerve impulse transmission which makes supplementation of downer cows with potassium a rational therapeutic measure (2, 11, 22).

Survival percentage of downer cows is generally poor and has been reported to be between 16 to 50% (14, 21). Any intervention that improves outcomes would be welcomed. In this study, 16 of the 19 downer cows (84%) survived following treatment with the dipotassium phosphate boluses.

It is believed that both persistent hypophosphatemia and hypokalemia contribute to prolonged recovery (2, 22, 23) and the dipotassium phosphate bolus addresses these deficiencies. In this study, it was shown that the bolus increased serum phosphorous in all five treated animals that were analyzed. The literature supports that oral administration of phosphate and potassium is the method of choice for treatment of clinical hypophosphatemia and hypokalemia (2, 4, 11). As noted previously, this field study had inherent limitations by using historical response rates to downer cows, but the high survival rate in animals treated with dipotassium phosphate boluses was a remarkable finding and warrants additional research.

### 4.3 Treatment of ketosis

Ketosis occurs in early lactation when energy demands exceed dietary energy intake with the mobilization of adipose tissue (2). Early treatment is beneficial for positive clinical outcomes and economic returns (2, 3). The most common clinical sign of hypophosphatemia is reduced feed intake (4, 5) which may initiate or exacerbate the clinical ketosis. Oral phosphate is an appetite stimulant (5, 8) and therefore has the potential to improve energy intake and may therefore be valuable in the treatment of ketosis. This study suggests that oral supplementation with dipotassium phosphate boluses results in an improved recovery from clinical ketosis as indicated by increasing rumination rate and milk production. The suggested mechanism of action was increased feed intake due to appetite stimulation by phosphate supplementation, thereby correcting the energy imbalance. It has been shown that the most common clinical sign of hypophosphatemia is depressed feed intake (24).

Decrease in feed intake that occurs in clinical ketosis can also result in hypokalemia (10–12, 25). The provision of supplemental potassium may also have acted to stimulate gastrointestinal motility and appetite stimulation, thereby improving the recovery rate from clinical ketosis.

### 4.4 Post-partum treatment with K Phos-Boost boluses

Dairy cattle are extremely vulnerable to the development of metabolic disorders in the post-partum period where levels of macro minerals and glucose are rapidly changing. Over 50% of dairy cattle are deficient in phosphorous during the early post-partum period (1, 4, 5) when metabolic disease such as hypocalcemia and ketosis are common (3). Metabolic health is also important for decreasing the risks of infectious diseases such as mastitis and metritis (3). This study attempted to demonstrate a beneficial effect of post-partum treatment with dipotassium phosphate boluses on metabolic health and disease prevention. Although we were unable to demonstrate a statistical reduction of subclinical ketosis and health events, there was a tendency toward a beneficial effect in milk production in second-lactation cows in the first 30 days in milk (*P* = 0.09) in dipotassium phosphate treated cows which may indicate an improvement in metabolic health in this group of cattle. Further research with larger sampling sizes is recommended. This study provides the variances that are necessary to perform power calculations required for future study design.

## 5 Conclusion

The dipotassium phosphate bolus is a novel, easy to administer product and has the potential to treat and prevent several important metabolic diseases in dairy cattle. The studies described in this paper are early investigations and further research will be required to further demonstrate the applications of a dipotassium bolus in dairy cows.

## Data availability statement

The raw data supporting the conclusions of this article will be made available by the authors, without undue reservation.

## Ethics statement

The animal studies were approved by Alberta Veterinary Laboratories' Animal Ethics Committee. The studies were also reviewed and approved by Alberta Veterinary Laboratories' field veterinarian in consultation with the practicing veterinarian associated with the study site. The studies were conducted in accordance with the local legislation and institutional requirements. Written informed consent was obtained from the owners for the participation of their animals in this study.

## Author contributions

WV: Conceptualization, Formal analysis, Investigation, Methodology, Project administration, Writing—original draft, Writing—review & editing. SZ: Conceptualization, Formal analysis, Investigation, Methodology, Project administration, Writing—original draft, Writing—review & editing. JR: Data curation, Formal analysis, Methodology, Visualization, Writing—original draft, Writing—review & editing. KB: Data curation, Formal analysis, Investigation, Methodology, Visualization, Writing—review & editing. MO: Conceptualization, Formal analysis, Funding acquisition, Investigation, Methodology, Project administration, Resources, Supervision, Writing—original draft, Writing—review & editing.
