# Supplementary material for: Evaluation of a novel dipotassium phosphate bolus for treatment of metabolic disorders in dairy cattle
Source: Front Vet Sci. 2023 Dec 8;10:1274183. doi: 10.3389/fvets.2023.1274183 (PMC10748419; doi:10.3389/fvets.2023.1274183)
Supplement: Supplementary file 1 [file Data_Sheet_1.PDF]

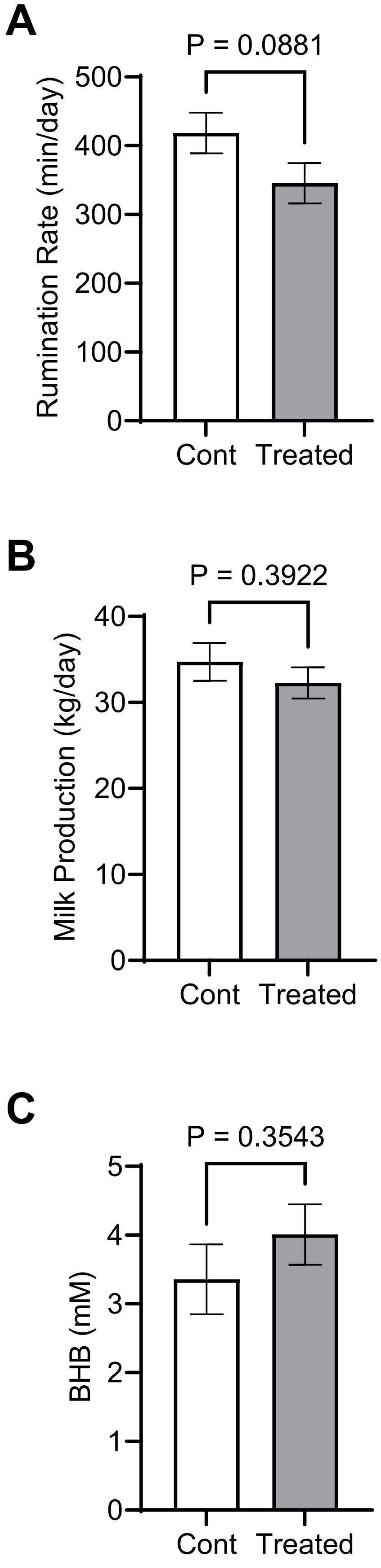

Figure S1. Outcomes at Day 0 (i.e., prior to bolus treatment) for animals allocated to the untreated (Control) versus K Phos-Boost bolus (Treated) groups. (A) Rumination Rate (min/day), **(B)** Milk Production (kg/day), **(C)** Serum Beta-Hydroxybutyrate (mM). Bars represent the mean ± SEM (Standard Error of the Mean). For panel (A), *n*=22 (Control) or 27 (Treated); for panel (B), *n*=23 (Control) or 29 (Treated) animals. A subset of those animals were examined for BHB (panel C), such that *n*=14 (Control) or 24 (Treated). Data were analyzed for statistical significance using an unpaired, two-tailed T-test.
